# Interleukin-4 Programmed Macrophages Suppress Colitis and Do Not Enhance Infectious-Colitis, Inflammation-Associated Colon Cancer or Airway Hypersensitivity

**DOI:** 10.3389/fimmu.2021.744738

**Published:** 2021-10-06

**Authors:** Blanca E. Callejas, Graham A. D. Blyth, Nicholas Jendzjowsky, Arthur Wang, Anshu Babbar, Konstantin Koro, Richard J. A. Wilson, Margaret M. Kelly, Eduardo R. Cobo, Derek M. McKay

**Affiliations:** ^1^ Gastrointestinal Research Group, Inflammation Research Network and Host-Parasite Interaction Group, Department of Physiology and Pharmacology, Calvin, Phoebe & Joan Snyder Institute for Chronic Diseases, Cumming School of Medicine, University of Calgary, Calgary, AB, Canada; ^2^ Department of Microbiology, Immunology and Infectious Disease, Cumming School of Medicine, University of Calgary and Production Animal Health, Faculty of Veterinary Medicine, University of Calgary, Calgary, AB, Canada; ^3^ Department of Physiology and Pharmacology, Hotchkiss Brain Institute, Cumming School of Medicine, University of Calgary, Calgary, AB, Canada; ^4^ Production Animal Health, Faculty of Veterinary Medicine, University of Calgary, Calgary, AB, Canada; ^5^ Department of Pathology and Laboratory Medicine, Calvin, Phoebe & Joan Snyder Institute for Chronic Diseases, Cumming School of Medicine, University of Calgary, Calgary, AB, Canada; ^6^ Alberta Children’s Hospital Research Institute, Cumming School of Medicine, University of Calgary, Calgary, AB, Canada

**Keywords:** intestinal inflammation, macrophage-based therapy, colitis-associated colorectal cancer, *Citrobacter rodentium*, airway inflammation

## Abstract

The murine interleukin-4 treated macrophage (MIL4) exerts anti-inflammatory and pro-healing effects and has been shown to reduce the severity of chemical-induced colitis. Positing M(IL4) transfer as an anti-inflammatory therapy, the possibility of side-effects must be considered. Consequently, bone marrow-derived M(IL4)s were administered *via* intraperitoneal injection to mice concomitant with *Citrobacter rodentium* infection (infections colitis), azoxymethane/dextran sodium sulphate (AOM/DSS) treatment [a model of colorectal cancer (CRC)], or ovalbumin sensitization (airway inflammation). The impact of M(IL4) treatment on *C. rodentium* infectivity, colon histopathology, tumor number and size and tissue-specific inflammation was examined in these models. The anti-colitic effect of the M(IL4)s were confirmed in the di-nitrobenzene sulphonic acid model of colitis and the lumen-to-blood movement of 4kDa FITC-dextran and bacterial translocation to the spleen and liver was also improved by M(IL4) treatment. Analysis of the other models of disease, that represent comorbidities that can occur in human inflammatory bowel disease (IBD), revealed that M(IL4) treatment did not exaggerate the severity of any of the conditions. Rather, there was reduction in the size (but not number) of polyps in the colon of AOM/DSS-mice and reduced infectivity and inflammation in *C. rodentium*-infected mice in M(IL4)-treated mice. Thus, while any new therapy can have unforeseen side effects, our data confirm and extend the anti-colitic capacity of murine M(IL4)s and indicate that systemic delivery of one million M(IL4)s did not exaggerate disease in models of colonic or airways inflammation or colonic tumorigenesis.

## Introduction

Typically associated with the phagocytosis of microbes, macrophages perform a myriad of functions in host defense and homeostasis. This versatile cell is highly responsive to its’ microenvironment and has been broadly classified into (1): classically activated macrophages (CAMs) that are considered pro-inflammatory and are evoked by exposure to microbial stimuli and interferon-γ (IFNγ); and (2), alternatively activated macrophages (AAM) elicited by, for example, cytokines, apoptotic bodies and immune complexes (1, 2). The AAM (or regulatory macrophage) exerts anti-inflammatory effects and promotes tissue repair and remodeling, angiogenesis, and wound-healing that involves interaction with fibroblasts (3, 4). Murine and human AAMs have the capacity to induce regulatory T-cells (5, 6), suggesting important roles in regulating adaptive immunity and the creation of an immunoregulatory environment.

The demonstration of the murine AAM’s ability to promote tissue repair, notably in the skin, but also the heart, kidney, and spinal cord, raises the possibility that these cells could be used for cellular immunotherapy (7–11). Prominent among AAMs is the IL-4 (± IL-13) treated macrophage M(IL4). We showed that systemic delivery of *in vitro* differentiated murine M(IL4)s significantly reduced the severity of dinitrobenzene sulphonic acid (DNBS), oxazolone, and dextran sodium sulphate (DSS) induced colitis in mice: in contrast, the administration of CAMs differentiated *in vitro* with IFNγ [or control M(0)] did not impact the outcome of the colitic disease (12–14). This anti-colitic effect of murine M(IL4)s and other AAM phenotypes was subsequently supported by other studies (15–19). In accordance with these findings, it was recently shown that soluble mediators from human M(IL4)s stimulated epithelial (i.e. monolayers of the human colon-derived T84 cell line) wound repair in an *in vitro* assay and, that these cells inhibited DNBS-induced colitis in *rag1^-/-^* mice (20).

Considerable proof-of-concept data for M(IL4)s as an anti-colitic therapy are available; however, with any new therapy, there is the possibility of side-effects. For instance, would the M(IL4)s pro-healing effect, if uncontrolled, result in fibrosis? While not identical, the M(IL4) and other AAMs share some similarities with myeloid-derived suppressor cells (MDSC) and tumor-associated macrophages (TAMs), raising the possibility that M(IL4)s could promote or exaggerate tumorigenesis (21). It has also been suggested that in a TH2-type environment, AAMs could increase an individual’s susceptibility to microbial infection (22).

Cognizant of these possibilities, the current study was designed to determine if murine M(IL4)s would affect the severity of disease in models of inflammation-associated colon cancer (CRC), infectious colitis and antigen-driven airways hypersensitivity. All of these conditions can be comorbidities in human inflammatory bowel disease (IBD), the target condition for M(IL4) treatment. The data herein, confirm and extend the anti-colitic effect of intraperitoneal delivery of M(IL4)s, and reveal that these cells, under the paradigms tested, did not boost tumor development in the azoxymethane (AOM)/DSS model of CRC, or immune cell infiltration and lung histopathology in the ovalbumin model of airway hypersensitivity. Intriguingly, inflammation in *Citrobacter rodentium*-infected mice was reduced by M(IL4) co-treatment. These proof-of-principle findings lend support for M(IL4)s as an anti-colitic therapy, and if translatable to human, suggest that M(IL4) treatment side-effects may be limited and would not offset the therapeutic benefit of this cellular immunotherapy.

## Materials And Methods

Animal experiments were approved by the University of Calgary Animal Care Committee in compliance with the guidelines of the Canadian Council on Animal Care and administered under protocol AC17-0115.

### Differentiation of M(IL4)s

Following a published protocol (13), bone marrow cells were isolated from femurs and tibias of C57BL/6J or BALB/c male mice (8-10 weeks old; Charles River Laboratories, Quebec, Canada). Cells were differentiated into macrophages by culture for 7 days in RPMI-1640 medium (Sigma-Aldrich) supplemented with 2% Pen/Strep, 1× GlutaMAX™, 20% fetal bovine serum (FBS) (all Gibco/Thermo Fisher Scientific) and 20 ng/mL recombinant macrophage-colony stimulating factor (M-CSF) (R&D Systems Inc.), changing medium on day 2 and day 5. Macrophages were then polarized with murine recombinant IL-4 (20 ng/mL; 48h; Cedarlane Labs, Mississauga, Ontario, Canada). Conversion to an M(IL4) was tested by qPCR analysis of CD206, FIZZ1, Ym1, arginase-1 (Arg-1) and CD14 mRNA expression (See **Supplementary Table 1** for PCR primer sequences) (13). We have previously shown that bone marrow-derived macrophages from BALB/c and C57BL/6J mice responded similarly to 20 ng/mL of IL-4 with up-regulation or arginase-1, FIZZ1 and Ym1 (12–14); only batches of M(IL4)s that showed this phenotypic response were used in the following studies.

### Induction of DNBS-Colitis and Assessment

Male BALB/c mice (8-10 weeks old) received an intraperitoneal (ip.) injection of M(IL4)s (1×10^6^ in 500 μL PBS), 48h before intra-rectal instillation of DNBS (3 mg in 100 µL 1:1 PBS/ethanol solution; MP Biomedicals, Santa Ana, CA) (12) [M(0) or M(IFNγ)-treated cells were not used as a comparator cell type in these studies because, as noted, neither phenotype was anti-colitic (12–14) and the present study was designed specifically to address the issue of putative side effects of administration of a therapeutic macrophage, i.e. M(IL4)]. Animals were monitored daily, weight recorded, and on humane euthanization, 72h after DNBS a macroscopic disease activity score was calculated on a 5-point scale based on weight change, evidence of watery/bloody diarrhea, colon length and macroscopic ulceration. A portion of mid-colon was immersion fixed in 10% neutral-buffered formalin (NFB) for 72h, then paraffin-embedded, and sections (5 µm) stained with hematoxylin and eosin (H&E) and histopathology scored in a blinded fashion on a 12-point scale (12). Additional sections of the colon were de-waxed, rehydrated and then treated with anti-Ly6G antibodies (1:100, 16-9668-85; eBioscience) to identify neutrophil infiltration (see immunostaining protocol below) (23).

To assess epithelial barrier function, mice were gavaged with 100 µL of 50 mg/mL FITC-dextran solution (FITC-4-kDa dextran; Sigma-Aldrich) 3h before being euthanized. Serum was collected prior to necropsy, fluorescence intensity measured (excitation, 492 nm; emission, 525 nm), and FITC-dextran concentrations read off a standard curve (24). Additionally, the spleen and liver were excised under sterile conditions, weighed, and homogenized in 3 mL of sterile PBS. Ten and 100 µL of the homogenate were inoculated onto Luria broth (LB) agar plates and incubated at 37°C for 48h in aerobic conditions. Subsequently, colony forming units (CFUs) were counted, corrected for sample dilution and are expressed as CFU/100 mg tissue (24).

As an indicator of local inflammation, segments of the colon were weighed, homogenized, and suspended in either hexadecyltrimethylammonium bromide (HTAB) buffer (50 mg tissue wet-weight/mL) for myeloperoxidase (MPO) activity or in sterile-PBS for measurement of cytokines. After homogenization, supernatants were collected and MPO activity was determined as before and represented as units U in 100 mg of tissue, where 1 U equals the amount of MPO required to degrade 1 µM H_2_O_2_/min at room temperature (12). Tissues homogenized in sterile-PBS were assessed for levels of IL-1β, TNF-α, IL-10 and CXCL1 by ELISA following the manufactures instructions (PeproTech, CranBury, NJ) (25).

### *Citrobacter rodentium* Infection and Assessment

C57BL/6J mice (8-10 weeks old, originally purchased from The Jackson Laboratory housed for several generations in a pathogen-free environment at the Univ. Calgary) were injected with M(IL4)s (1×10^6^ in 500 μL PBS, ip.). Two days later, mice were orally gavaged with *C. rodentium* (strain DBS 100; 5×10^8^ CFU in 200 µL PBS) (26, 27). Feces were collected at 3-, 5-, and 7-days post-infection (dpi), homogenized at 0.1g/1 mL of sterile PBS, then serial dilutions were added to MacConkey agar plates. After 24h of aerobic culture at 37°C, colonies were counted to obtain bacterial CFU/g (26). Mice were euthanized at 7 dpi, colon length measured, and segments of mid-colon processed for H&E staining. Fecal lipocalin-2 was measured as a general marker of inflammation by ELISA (R&D Systems) and following the provided manufactures instructions (26, 27).

Total RNA was isolated from colon tissues (Aurum Total RNA Mini Kit, Bio-Rad Laboratories, Hercules, CA), quantified (Nanodrop 1000 Spectrophotometer, Thermo Fisher Scientific, Wilmington, DE), and 0.5 μg of RNA converted to cDNA using iScript kit (Bio-Rad Lab, Canada). Quantitative real-time polymerase chain reaction (qPCR) was performed with SYBR Green Supermix (Bio-Rad, Cat. # 1725274), which consisted of 40 amplification cycles per run (26) using primer sequences shown in **Supplementary Table 1** and normalized to the housekeeping gene 18S rRNA. Then the relative quantitative target gene expression of treatment groups was calculated by using 2^-ΔΔCt ^method using control group as calibrator samples. Reactions were run in triplicate (the same process was applied to assess mRNA in bone marrow-derived macrophages).

To assess cellular composition of the colon, the distal 50% of the colon was opened longitudinally and incubated thrice for 15 min each in Hanks’ Balanced Salt Solution (HBSS) with 10% FBS and 2 mM EDTA at 37°C to remove epithelial cells. After each incubation step, tubes were shaken for 10 seconds and medium containing epithelial cells was discarded. The lamina propria cells were centrifuged at 400*xg* for 10 min at 4°C, and the pellet re-suspended in PBS (26). The cells were stained with antibodies against CD45 (563891; BD Biosciences), CD3e (557984; BD Biosciences), CD4 (561104; BD Biosciences), Ly6G (561104; BD Biosciences) and CD17a (506915; BioLegend) for 30 min at 4°C at the concentrations shown in **Supplementary Table 2**. The cells were washed twice with PBS/BSA/2mM EDTA and analyzed on a FACS CANTO-II (BD BioSciences).

### Colorectal Cancer Induction and Assessment

Azoxymethane (AOM: Sigma, MO) + dextran sulphate sodium (DSS; MW 40,000, Alfa Aesar, Tewksbury, MA) is a common model of CRC (23, 28). Male C57BL/6J mice (8-10 weeks old) received AOM (12.5 mg/Kg, ip.) and five days later, 2% DSS in drinking water for 7 days *ad libitum* followed by 14 days of regular water. This 7-day DSS-water + 14-day regular water regimen was repeated for two more cycles and mice were euthanized on day 113. One group of mice received M(IL4)s (1×10^6^, ip. in 500 μL PBS) 33 days after AOM (i.e. early) or 54 days after AOM injection. Upon euthanization, the colon was removed and opened longitudinally, polyps counted and measured, and tissues collected for H&E staining (assessed in a blinded fashion by K. Koro), immunodetection of vimentin and E-cadherin, qPCR and for tissue levels of cytokines by ELISA.

Paraffin-embedded sections (5 µm) of the colon were de-waxed, rehydrated and then treated with anti-vimentin (1:100, 3932S; Cell Signaling) or anti-E-cadherin (1:300, 610181; BD eBioscience) antibodies overnight at 4°C. Following washing, matched secondary antibodies were applied. For immunohistochemistry, the goat anti-rat-HRP conjugated was incubated for 30 min at room temperature (RT) (1;500, 405405, Biolegend). To visualize positive staining cells, tissue sections were stained with diaminobenzidine (DAB) (ab64238; Abcam) and counterstained with hematoxylin. For immunofluorescence goat anti-mouse Alexa fluor 488 (1:500, A11029; Invitrogen) and goat anti-rabbit Alexa fluor 594 (1:1000, A11005; Invitrogen) were incubated for 2h at RT and after incubation, the nuclei were stained with DAPI at 1:1000 for 5 min (23). Representative images were captured on an Olympus BX41 microscope fitted with a U-TMAD T mount adapter, using cell Sens software (Olympus).

### Ovalbumin Sensitization and Airway Assessment

Male BALB/c mice (8-10 weeks old) were immunized with ovalbumin 50 µg (A5503; Sigma) and 1.5 mg aluminum hydroxide (AC219130250; Fisher) dissolved in 100 µL PBS (ip.) on days 1, 2 and 3. On days 15-18 (inclusive) mice were exposed to 20 min of 5% nebulized OVA dissolved in PBS in an airtight chamber (29). Mice received 1x10^6^ M(IL4) (ip.) 2 days prior to the first dose of aerosolized OVA, and airway inflammation was assessed on day 19.

Bronchoalveolar lavage fluid (BALF) was collected from all mice. With the upper trachea cannulated, lungs were lavaged (1 mL per lavage x 3) with 0.9% NaCl. Cells in the bronchoalveolar fluid were sedimented by centrifugation (20 min at 4500*xg*, 4°C) and re-suspended in PBS. A 100 µL sample of BALF was centrifuged (Shandon Cytospin 4 cytocentrifuge, Thermo Scientific, Waltham, MA, 6 min at 4500*xg*) and cells collected on non-coated glass slides, fixed in 95% ethanol and H&E stained. Total leukocytes were determined by hemacytometer counting and identification of 200 cells was completed according to standard morphologic criteria (30).

To assess lung pathology, the left lung was inflated with 10% NBF and fixed for 72h, processed to paraffin and 5 µm sections were collected on coded slides, stained with H&E and scored in a blinded fashion (M. Kelly) using a published scale (31). The right lung was used to evaluate collagen levels by analysis of hydroxyproline using a commercial assay according to the manufacturer’s instructions (K218, BioVision). Briefly, lungs were dried, weighed, and homogenized in 100 μL ddH_2_O for every 10 mg of tissue; 100 μL of concentrated 6N HCl were added to homogenized sample. Hydrolyzed samples were incubated at 120°C for 3 hours and then 30 μL of each hydrolyzed sample transferred to a 96-well plate and 100 μL of the Chloramine T reagent added to each sample and standard and incubated at room temperature for 10 min. Finally, 100 μL of the DMAB reagent was added to each well and incubated for 90 min at 60°C. The amount of collagen generated was determined from a collagen calibration curve. The absorbance measured at 560 nm of each hydrolyzed sample (13).

### Statistical Analysis

Data are presented as the mean ± standard error of the mean (SEM). Data were analyzed and graphed using GraphPad Prism 6 (GraphPad Software). Unless otherwise stated, data were analyzed using a one-way analysis of variance (ANOVA) and when p<0.05, pairwise comparisons of the means were examined with Tukey’s test for parametric data. The Kruskal-Wallis test followed by Dunn’s multiple comparison test was applied to nonparametric data.

## Results

### M(IL4) Transfer Protects Against Experimental Colitis

**Figure 1:**
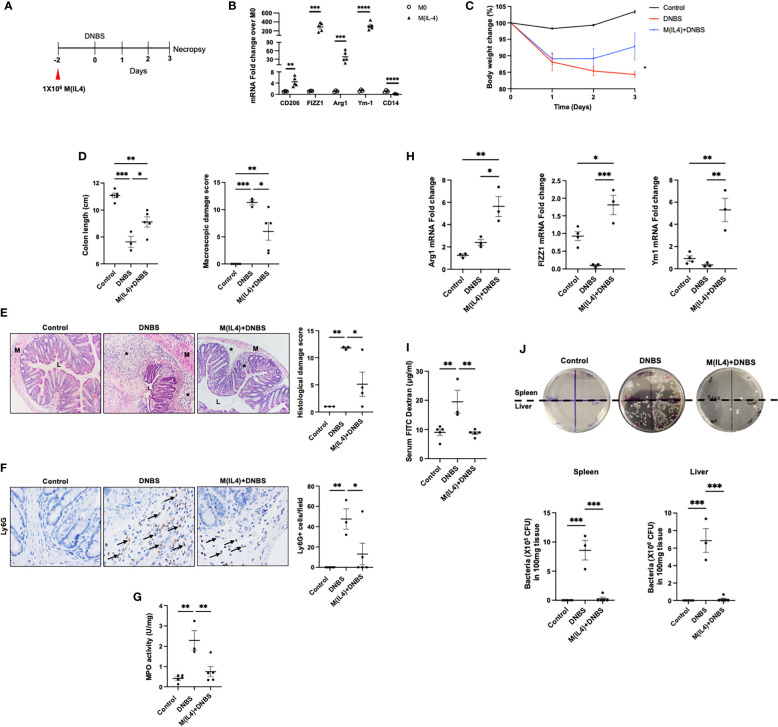
M(IL4)s protect against murine colitis. Male BALB/c mice were injected with M(IL4)s (1×10^6^, ip.) 48h prior to intra-rectal delivery of DNBS (3 mg in 100 µL 50% ethanol) **(A)**. Murine bone marrow-derived macrophages were treated with IL-4 (20 ng/mL per 2.5×10^5^ cells/mL) for 48h and conversion to an M(IL4) tested by expression of CD206, FIZZ1, Arg-1, Ym-1 and CD14 mRNA **(B)**. During induction of colitis, weight was recorded daily **(C)**. On necropsy at 72h post-DNBS, colon length was recorded, and a macroscopic disease activity score calculated **(D)**. Panel **(E)** shows representative H&E images (original magnitude, x20) and histopathology scores. Panel **(F)** shows representative images of Ly6G staining (original magnitude, x40) and enumeration of Ly6G^+^ cells/high power field of view (n = 3-4 mice). Myeloperoxidase (MPO) activity in colonic extracts as a measure of predominantly neutrophil infiltration is shown in panel **(G)**. Panel **(H)** depicts qPCR data of arginase -1 (Arg1), FIZZ1 and Ym1 in colon extracts. Epithelial barrier function, as assessed by lumen-to-blood movement of 4kDa FITC-dextran and bacterial translocation to the spleen and liver is shown in panels **(I, J)** (data are means ± SEM; n=5 mice; one-way ANOVA followed by Tukey’s test for parametric data and Dunn multiple comparison test for nonparametric data; *p < 0.05, **p < 0.01, ***p < 0.001, ****p ≤ 0.0001; M, outer layers of muscle; L, gut lumen; * inflammatory infiltrate).

To test the anti-colitic effect of M(IL4) transfer, macrophages from BALB/c mice were differentiated *in vitro* with IL-4 and injected (ip.) into mice two days before induction of colitis with DNBS (**Figure 1A**). qPCR-analysis revealed increased CD206, FIZZ1, Ym1, arginase-1, and decreased CD14 mRNA expression confirming the identity of the murine M(IL4) (14) (**Figure 1B**). Mice treated with DNBS displayed a drop in body weight, a shorter colon, increased macroscopic disease scores (based on ulceration, lack of consistency in feces, and bleeding) and significant histopathology (**Figures 1C** **–E**). Consistent with previous findings in this model (13), mice treated with M(IL4)s had significantly less severe DNBS-induced colitis (**Figures 1C** **–E**), a range of benefit consistent with prior investigations (12–14), and these observations are supported by significantly reduced infiltration of Ly6G^+^ cells and colonic MPO levels in tissues from M(IL4)+DNBS treated mice compared to tissue from DNBS-only treated mice (**Figures 1F, G**). qPCR analysis revealed increased expression of arginase-1, FIZZ1 and Ym1 in colon extracts from mice administered M(IL4)s (**Figure 1H**). While these data suggest localization of the transferred M(IL4)s to the colon, and are consistent with our prior immunodetection studies (14), they do not negate the possibilities that host resident macrophages up-regulated the expression of these genes or that other cells in the colon are responsible for, or contribute to, the increased arginase-1, FIZZ1 and Ym1.

To characterize the protective effects of M(IL4) on the epithelial layer in DNBS-treated mice, gut permeability was evaluated by orally administering FITC-dextran to mice. Measurement of FITC-dextran in the serum of DNBS-treated mice revealed a decrease in epithelial barrier function, that was not observed in DNBS+M(IL4)-treated animals (**Figure 1I**). Furthermore, the increased translocation of gut bacteria to the spleen and liver that occurred in DNBS-treated mice was significantly reduced in mice co-treated with M(IL4)+DNBS (**Figure 1J**). Collectively, these results confirm the protective role of M(IL4) in the DNBS-model of acute colitis.

### M(IL4) Reduces *C. rodentium*-Induced Colitis

**Figure 2:**
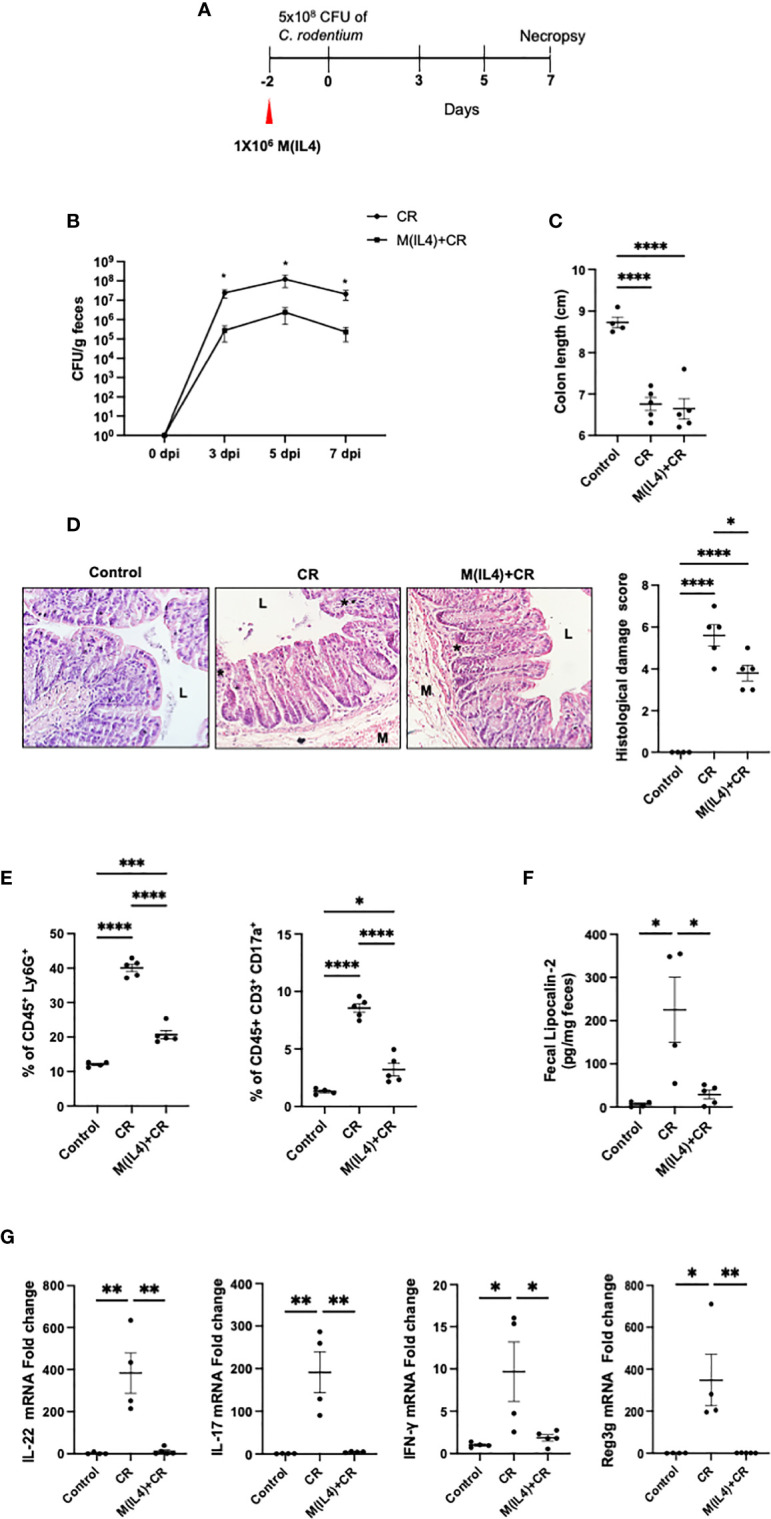
M(IL4)s reduce colonic inflammation in mice infected with *C rodentium*. Male C57BL/6J mice were injected with M(IL4)s (1×10^6^, ip.) and 48h later received 5×10^8^ CFU of *C rodentium* (CR) by oral gavage **(A)**. Shedding of *C rodentium* was monitored in the feces at 3, 5, and 7 day post-infection (dpi) **(B)** and on necropsy, colon length was measured **(C)** and tissue processed for histopathology scoring **(D)**; representative H&E sections shown (original mag., x2). Panel **(E)** shows colonic levels of Ly6G^+^ and IL17^+^ cells as determined by flow cytometry. Lipocalin-2 levels in feces collected at 7 dpi are shown in Panel **(F)**. Real-time qPCR analysis of colonic IL-22, IL17, IFN-γ, and Reg3g mRNA **(G)** (data are means ± SEM; n = 4-5 mice; one-way ANOVA followed by Tukey’s test for parametric data and Dunn multiple comparison test for nonparametric data; *p < 0.05, **p < 0.01, ***p < 0.001, ****p ≤ 0.0001; M, outer layers of muscle; L, gut lumen; * inflammatory infiltrate).

It has been suggested that M(IL4)s might predispose to bacterial infection (22). To assess this, and for comparison with chemical-induced colitis, the effect of M(IL4) transfer (C57/BL6 mice) in the *C. rodentium* model of infectious colitis was examined (27) (**Figure 2A**). As an index of infection, fecal shedding of *C. rodentium* was lesser in M(IL4)-treated mice compared with untreated mice at 3, 5 and 7 days dpi (~100-fold reduction) (**Figure 2B**). While the shortening of the colon was not affected by M(IL4)-treatment (**Figure 2C**), this treatment did result in a mild, but statistically significant reduction in colonic histopathological damage and reduced neutrophils (Ly6G^+^) and Th17 cell infiltrations in the colon (**Figures 2D, E**). The M(IL4)-treatment also reduced fecal levels of lipocalin-2 (**Figure 2F**) and colonic expression of IL-22, IL-17, IFN-γ and Reg3g mRNA (**Figure 2G**) in *C. rodentium*-challenged mice. Overall, these findings indicate that M(IL4) transfer protects against colitis induced by *C. rodentium*-infection.

### M(IL4) Transfer Does Not Influence the Progression of Colorectal Cancer

**Figure 3:**
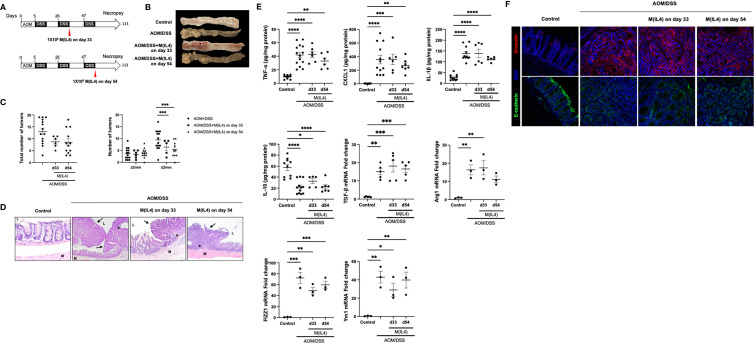
M(IL4)s do not exaggerate colitis-associated colon cancer. Male C57BL/6J mice were treated as shown in panel **(A)** (azoxymethane (AOM) 12.5 mg/Kg ip. and 2% (wt./vol.) dextran sodium sulphate (DSS) in drinking water for 7 days), with M(IL4)s (1×10^6^, ip.) given in two paradigms. On necropsy at day 113, colons were photographed and tumors counted and measured **(B, C)**, and tissue processed for histological assessment, with representative H&E images shown in **(D)** (original mag., x20; M, muscle; L, lumen; arrow, neoplastic lesions; *, mixed lymphocyte, plasma cell, and neutrophil infiltrate). Panel **(E)** shows colonic levels of cytokines (mRNA for TGFβ) and qPCR data of arginase -1 (Arg1), FIZZ1 and Ym1 in colon extracts. Panel **(F)** shows representative images of mid-colonic tissue immunostained for vimentin or E-cadherin (DAPI as nuclear stain) (data are means ± SEM; one-way ANOVA followed by Tukey’s test, *p < 0.05, **p < 0.01, ***p < 0.001, **** p ≤ 0.0001).

Since macrophages activated by TH2-type cytokines share some characteristics of tumor-associated macrophages (TAMs) (32) we evaluated the putative tumorigenic property of M(IL4) transfer in a model of colitis-associated CRC. Two experimental paradigms were assessed: mice received M(IL4)s from C57/BL6 mice 33 days after the azoxymethane (AOM) or later in the progression of the CRC at 54 days after AOM treatment, and mice were humanely euthanized at day 113 (**Figure 3A**).

Systemic delivery of M(IL4)s at an early (day 33) or later (day 54) time-point in the progression of AOM/DSS-evoked CRC did not affect the number of macroscopically observable tumors, but did significantly reduce the number of tumors of >2 mm diameter by ~50% (**Figures 3B, C**). Blinded histological examination of colon demonstrated features of inflammation and neoplastic transformation (i.e. adenomas and adenocarcinoma). Background colonic mucosa inflammation was characterized by ulceration, neutrophilic cryptitis, hyperplastic/regenerative mucosa changes, and mixed lymphoplasmacytic inflammatory infiltrate. Neoplastic lesions showed altered cytology, including mucin depletion, nuclear stratification, increased nuclear/cytoplasmic ratio, and nuclear hyperchromasia. However, there were no significant microscopically identifiable differences between the degree of inflammation or types and configuration of adenomas between AOM/DSS-treated mice ± M(IL4) at day 33 or day 54 groups (**Figure 3D**). In initial studies, M(0)s were used as a control group but the course of the CRC was not affected (total number of tumors; AOM/DSS = 13 ± 1 versus AOM/DSS+M(0) d33 = 13 ± 2 and AOM/DSS+M(0) d54 = 12 ± 3: number of tumors >2mm diameter, AOM/DSS = 9 ± 1, AOM/DSS+M(0) d33 = 11 ± 1, AOM/DSS+M(0) d54 = 11 ± 1, mean ± SEM, n=3-4).

Analysis of TNF-α, CXCL1, IL-1β, IL-10 protein and TGF-β mRNA levels in colonic tissue segments devoid of obvious adenomas revealed the expected increases in these cytokines in the AOM/DSS mice were not affected by M(IL4) treatment (**Figure 3E**). On necropsy, qPCR of colonic extracts revealed increased mRNA for arginase-1, FIZZ1 and Ym1 in mice treated with AOM/DSS ± M(IL4); however, while different from controls, expression in tissue from mice receiving M(IL4)s was not different from that from the AOM/DSS only group (**Figure 3E**). Similarly, immunodetection of the distribution of vimentin and E-cadherin, as markers of epithelial-mesenchymal transition (EMT), revealed no clear differences between tissue sections from AOM/DSS ± M(IL4)-treated mice (**Figure 3F**). These data indicate that the transfer of M(IL4) in the early or later stages in this chemical model of CRC does not promote tumor progression.

### M(IL4) Transfer Does Not Exacerbate Airway Inflammation

**Figure 4:**
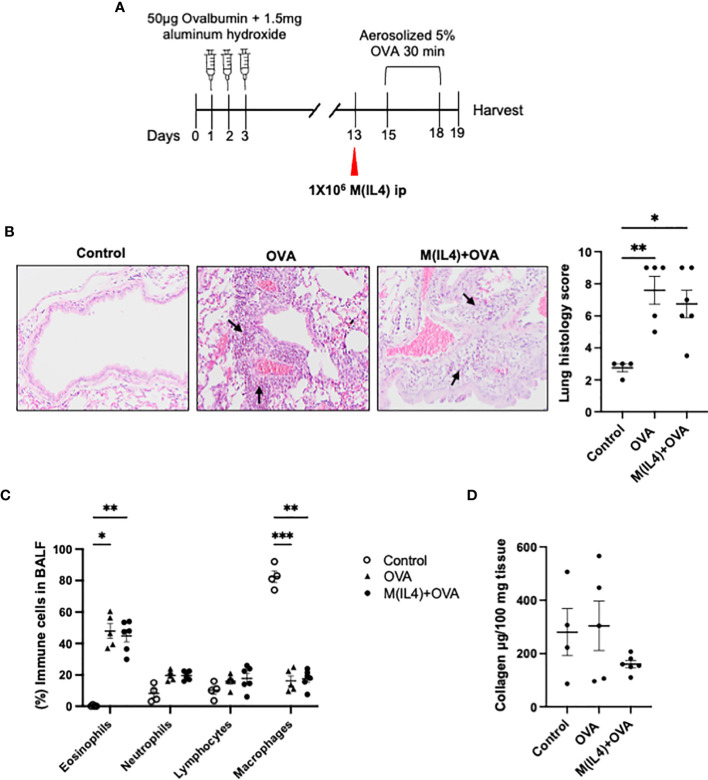
M(IL4)s do not exacerbate airway inflammation in OVA-induced asthma. Male BALB/c mice were treated as shown in panel **(A)**. Formalin-fixed lungs were assessed for histopathology using H&E sections (arrows, eosinophils infiltration) **(B)**. Percentage of immune cells was performed on H&E-stained cytospin of bronchoalveolar lavage fluid (BALF) **(C)**, and total lung collagen levels were measured by commercial colorimetric assay **(D)** (data are means ± SEM; n = 5; one-way analysis of variance followed by Tukey’s test; *p < 0.05, **p < 0.01, ***p < 0.001).

M2-macrophages have been presented as a concern in allergic asthma (33). In order to determine if M(IL4) transfer aggravates airways hypersensitivity, the OVA-sensitization model was employed. M(IL4)s from BALB/c mice were injected ip. 2-days before OVA challenge treatment (**Figure 4A**). In this classical model, OVA challenge of the sensitized mouse results in histopathology, remodeling of the lungs, and a substantial polymorphonuclear infiltration (mostly eosinophils but also some neutrophils): co-treatment with M(IL4)s had no appreciable effect on these aspects of the lung (**Figures 4B, C**). Analysis of lung collagen content revealed no significant increase in the OVA-challenged sensitized mice over controls and this was not affected by an M(IL4) treatment (**Figure 4D**). Thus, we have no evidence of M(IL4) aggravation of airway inflammation induced by OVA in sensitized and challenged mice.

## Discussion

The burden of IBD is increasing worldwide and despite the availability of new therapeutics, many patients receive minimal or no relief from these, and others become refractory to an effective treatment over time. Cellular therapy is an approach to auto-inflammatory disease in which, typically, mesenchymal stem cells or regulatory immune cells are used to blunt inflammation (34, 35). Expanding on the ability of macrophages to promote wound healing (3), we showed that murine M(IL4)s reduced disease in three models of chemical-induced colitis (12–14); a finding confirmed by others using M(IL4)s or other phenotypes of regulatory macrophage (15–19). All medications have side effects, and this provided the impetus to determine if the anti-colitic M(IL4) would exacerbate other conditions that can occur in IBD.

M(IL4)s in infectious colitis, inflammation-associated CRC and airways inflammation was assessed for two main reasons. First, macrophages are important in combating microbes, are linked to cancer progression (i.e. TAMs), and a type-2 macrophage can be pro-fibrotic in the lung (36). Second, some patients with IBD have airway inflammation, increased incidence of CRC, and altered susceptibility to infection such as the increased presence of pathobiont *E. coli* associated with Crohn’s disease (37–39). Systemic delivery of M(IL4)s *via* intraperitoneal injection did not aggravate the outcome in any of the models of disease, adding support for macrophages as a therapeutic target in IBD (20, 40).

Within the context of helminth-therapy for colitis, we noted increased expression of colonic FIZZ1 and arginase-1 mRNA, markers indicative of an M(IL4) (12); however, these markers are not exclusive to M(IL4)s. To negate any ambiguity, bone marrow-derived macrophages treated with IL-4 were shown to block colitis (13). Complementing this finding, the current study shows that M(IL4)-treated mice following challenge with DNBS had lesser accumulation of neutrophils in the colon and increased enteric barrier function compared to DNBS-only treated mice.

*C. rodentium* is a natural Gram-negative attaching/effacing pathogen in mice, with pathogenesis in the colon similar to enteropathogenic *Escherichia coli* (EPEC) in humans (27). Postulating that M(IL4)s might counter classically activated macrophages’ anti-microbial responses, mice were injected with M(IL4s) then challenged with *C. rodentium*. Rather than rendering the mice vulnerable to *C. rodentium*, mice treated with M(IL4)s showed enhanced protection against infection and less colonic histopathology that correlated with reduced mRNA expression of IL-22, IL-17 and IFNγ, hallmarks of *C. rodentium*-colitis. These data appear to contradict Weng *et al.* who reported that AAMs mediated the exacerbation of *C. rodentium* infection in mice infected with the parasitic helminth, *Heligmosomoides polygyrus* (22). However, while evidence of accumulation of AAMs in the co-infected mice was presented (IL-4 increases following *H. polygyrus-*infection), numerous factors in this *in vivo* setting could have affected AAM activity. Indeed, the bactericidal capacity of AAMs is not clear, with increased and decreased phagocytosis and killing of bacteria being reported (41–43). We speculate that this reflects the plasticity of the macrophage and potentially the confusion created by considering AAMs as a single group. Nevertheless, having confirmed the anti-colitic effect of the M(IL4)s used here, the murine M(IL4) was found to reduce the severity of inflammation induced by *C. rodentium*, suggesting that susceptibility to bacterial infection need not be a significant side effect of M(IL4) therapy.

Chronic inflammation is a hallmark of cancer and patients who suffer from ulcerative colitis are at higher risk of developing CRC (38). Macrophage association with tumors is well documented, and whether described as TAMs, AAM or M2-cells, the immunosuppressive nature of these cells *via* cognate ligands or the release of soluble signals has been linked to tumor progression by suppression of anti-tumor immunity (44). For example, the number of M2-macrophages, identified as CD68^+^CD163^+^ cells on tissue sections, correlated with the progression and invasion of CRC (45). So while the link with immunosuppressive macrophages and CRC is not disputed, these cells are not identical to *in vitro* differentiated M(IL4)s; yet, the similarity between M(IL4)s and TAMs [e.g. both make TGFβ (46)], raises the possibility that M(IL4)s could promote CRC. Exploring this with the AOM/DSS model of CRC, we find that M(IL4)s delivered early or later in the development of CRC did not increase the progression or severity of disease. While there was a reduction in the number of tumors >2mm diameter in M(IL4)+AOM/DSS treated mice, the general pattern was that systemic delivery of M(IL4)s had negligible impact on the outcome of CRC in this model system. However, the reduction in the number of >2 mm tumors is intriguing and may suggest less invasiveness (i.e. malignancy), or that transient suppression of inflammation following M(IL4) administration slowed polyp growth. Preliminary studies transferring M(0)s, revealed that this group of macrophages affected neither the progression of the CRC nor tumor size, implying specificity in the M(IL4) affect.

We have noted that a portion of ip.-delivered M(IL4)s migrate to the colon (14), but their longevity there is unknown. Examination of arginase-1, FIZZ1, and Ym1 mRNA revealed increased expression in tissues from AOM/DSS-treated mice, which also occurred, but not to a greater extent, in colon from the M(IL4) co-treated mice. The significance of these findings are not clear, and, as mentioned, arginase-1, FIZZ1 and Ym1 expression is not restricted to M(IL4)s. The data herein suggest that their expression alone does not blunt the development of CRC, indicating that the reduction in average tumor size seen in M(IL4)-treated mice is likely not dependent on arginase-1, FIZZ1 or Ym1. We speculate, if M(IL4)s suppress inflammation-associated CRC (i.e. fewer larger tumors) this may require the presence of the cells and could be accomplished with repeated M(IL4) administrations: a possibility worthy of testing in future analyses.

From the perspective of developing M(IL4) as a therapy, the lack of an effect of M(IL4) transfer in the AOM/DSS model is encouraging. In fact, in some types of cancer, pro-inflammatory mediators such as IL-22, TNF-α, MIP-3α, CXCL2, CXCL3 and other CXCR2 ligands indirectly contribute to tumor growth through the proliferation of epithelial cells (e.g. STAT3, NF-κB) (47–49). In the current study, the reduction of tumor size could be due to the regulation of proliferation through some inflammatory mediators not assessed/identified in the current study. Yet, we would be remiss if we failed to mention some caveats (1): AOM/DSS is only one model of CRC and other time-points of delivery in the regime were not considered although one would hypothesis that early delivery of M(IL4)s could be beneficial because of their anti-colitic ability (2); it is possible that in other models or in humans that *in vivo* factors [e.g. IgG_4_ (50)] could promote an oncogenic phenotype in the M(IL4)s; and (3), while functionally equivalent in some aspects, human and murine M(IL4)s are not identical and findings with murine cells do not dismiss the possibility that human M(IL4)s could promote CRC, metastasis or cancer in other organs.

The macrophages’ ability to promote collagen deposition is cause for concern in airways inflammation (51). Macrophage accumulation in the lung is characteristic of murine models of allergic asthma (33) (**Figure 4**) and the cells often bear the hallmarks of AAM/M(IL4)s (e.g. arginase-1^+^, FIZZ1^+^, Ym1^+^). Treatment with agents that block AAM activity can reduce the severity of disease in models of airway hypersensitivity/asthma (52, 53), although such studies cannot rule out non-macrophage effects of the drugs. In contrast, others suggest that arginase-1^+^ AAMs can be anti-fibrotic (54) and repeated treatments of M(IL4)s over a three-week period did not elicit increased collagen deposition in the lungs (liver, spleen or colon) of mice co-treated with DNBS (13). Intravenous delivery of helminth-evoked AAMs reduced airways inflammation (55), while intra-nasal administration of an arginase-1^+^/FIZZ1^+^ AAM obtained from antibiotic-treated mice increased inflammation in the airway (56): this study also showed that intra-nasal M(IL4) delivery evoked lung eosinophilia, while intraperitoneal injection of AAMs reduced eosinophilia in allergic asthma (52). Intermediate between studies indicating that AAMs can promote airways inflammation (53) and others suggesting a benefit of AAMs in allergic conditions, the present study showed *in vitro* differentiated M(IL4)s given prior to allergen challenge had no effect in an OVA-model of airways hypersensitivity. M(IL4)-treated mice did not display an increase in collagen indicating no propensity towards fibrosis in this acute model setting. A variety of chemical and allergen models of airways inflammation are available, and it will be important to determine if M(IL4)s affect the inflammation, fibrosis or lung function in these models (57).

We speculate that the lack of consensus on the role of AAMs in airways inflammation indicates the spectrum of activity within this cell population, and that clarity may arise by adherence to descriptive nomenclature as presented by Murray and colleagues (58). It seems that the route of AAM or M(IL4) delivery affects the pro-inflammatory versus anti-inflammatory outcome in the airways, and in this context, it is noteworthy that intraperitoneal injected M(IL4)s did not accumulate in mouse lungs over a 5 day time-course (13). Also, the role of the microbiota in modifying the outcome of macrophage transfer should not be overlooked (44). Thus, it will be important to monitor lung function in any adoptive macrophage treatment strategy (59).

Having demonstrated the anti-colitic effect of M(IL4)s and the preservation of gut barrier function, the current study yielded no data to suggest that M(IL4) therapy would exaggerate or leave an individual more vulnerable to concomitant infection, CRC or airways inflammation. These observational data are presented in support of M(IL4) cellular therapy and could be augmented by mechanistic studies to elucidate, for example, how the administration of M(IL4)s resulted in reduced polyp size in the AOM/DSS model of CRC. We recognize that the range of potential side effects of any new therapy is large and we have considered but three of these, and that further testing of M(IL4)s in variations of the models presented herein and other disease model systems is important to garner a more holistic view of the M(IL4) in disease. Given that sex-differences have been noted in disease, it will be important to broaden the assessment of putative M(IL4) side effects by performing experiments with female mice (and also young and old animals) in models of disease. Thus, continued research efforts are required to precisely elucidate the anti-colitic mechanism of M(IL4)s and to test M(IL4)s in other models of infection, cancer and inflammatory disease (e.g. cirrhosis) (60), and to complement such studies it will be essential to assess putative side effects of human M(IL4)s in suitable model systems (20).

## Data Availability Statement

The raw data supporting the conclusions of this article will be made available by the authors, without undue reservation.

## Ethics Statement

The animal study was reviewed and approved by University Calgary Animal Care Committee in compliance with the guidelines of the Canadian Council on Animal Care and administered under protocol AC17-0115.

## Author Contributions

BC, project design, data collection and analysis, manuscript writing & review. GB, data collection and analysis, manuscript review. NJ, data collection and analysis, manuscript review. AW, data collection and analysis, manuscript review. AB, data collection and analysis, manuscript review. KK, blinded tissue scoring, analysis and manuscript review. RW, manuscript review. MK, blinded tissue scoring, analysis and manuscript review. EC, manuscript review. DM, project design, grant support, analysis, manuscript writing and review. All authors contributed to the article and approved the submitted version.

## Funding

This work was supported by a Grant-in-Aid from Crohn’s Colitis Canada to DM. BC is supported by an Alberta Innovates-Health Sciences Post-doctoral Fellowship. NJ is supported by the Francis Family Foundation as a Parker B Francis Fellow. Work from the Cobo laboratory is supported by a Discovery Grant from the Natural Sciences and Engineering Council of Canada (NSERC), RGPAS-2017-507827. 

## Conflict of Interest

The authors declare that the research was conducted in the absence of any commercial or financial relationships that could be construed as a potential conflict of interest.

## Publisher’s Note

All claims expressed in this article are solely those of the authors and do not necessarily represent those of their affiliated organizations, or those of the publisher, the editors and the reviewers. Any product that may be evaluated in this article, or claim that may be made by its manufacturer, is not guaranteed or endorsed by the publisher.
