# Supplementary material for: Interleukin-4 Programmed Macrophages Suppress Colitis and Do Not Enhance Infectious-Colitis, Inflammation-Associated Colon Cancer or Airway Hypersensitivity
Source: Front Immunol. 2021 Oct 6;12:744738. doi: 10.3389/fimmu.2021.744738 (PMC8527087; doi:10.3389/fimmu.2021.744738)
Supplement: Supplementary file 1 [file DataSheet_1.docx]

Supplementary Material

# Supplementary Table 1. Primer sequences using in qPCR analysis.

| **Gene** | **Primer Sequences** |
| --- | --- |
| *Mrc1 (CD206)* | Forward: 5ʹ-ATGCCAAGTGGGAAAATCTG-3 ʹ  Reverse: 5ʹ-TGTAGCAGTGGCCTGCATAG-3 ʹ |
| *FIZZ1* | Forward: 5ʹ-GGATGACTGCTACTGGGTGT-3 ʹ  Reverse: 5ʹ-AGAAGCAGGGTAAATGGGCA-3 ʹ |
| *Ym-1* | Forward: 5ʹ-CAGTGCCATGGTCTCTACTCCT-3 ʹ  Reverse: 5ʹ-CTTTACGCATTTCCTTCACCAG-3 ʹ |
| *Arg-1* | Forward: 5ʹ-CTGAGCTTTGATGTCGACGG-3 ʹ  Reverse: 5ʹ-TCCTCTGCTGTCTTCCCAAG-3 ʹ |
| *CD14* | Forward: 5ʹ-GCTCTGCGTGTCTGCCCTCT-3 ʹ Reverse: 5ʹ-TCTGTGAAGCTGCCGGGAGGT-3 ʹ |
| *TGF-* *β* | Forward: 5ʹ-CCACCTGCAAGACCATCGC-3 ʹ  Reverse: 5ʹ-CTGGCGAGCCTTAGTTTGAC-3 ʹ |
| *18S* | Forward: 5ʹ-ATGGCCGTTCTTAGTTGGTG-3ʹ Reverse: 5ʹ-CGCTGAGCCAGTCAGTGTAG-3 ʹ |
| *Hprt* | Forward: 5ʹ-AGTCCCAGCGTCGTGATTAG-3 ʹ  Reverse: 5ʹ-TTTCCAAATCCTCGGCATAATGA-3 ʹ |
| *Il22* | Forward: 5ʹ-TTGAGGTGTCCAACTTCCAGCA-3 ʹ  Reverse: 5ʹ-AGCCGGACGTCTGTGTTGTTA-3 ʹ |
| *Il17* | Forward: 5ʹ-AACACTGAGGCCAAGGACTT-3 ʹ  Reverse: 5ʹ-ACCCACCAGCATCTTCTCG-3 ʹ |
| *Ifng* | Forward: 5ʹ-TGGCTCTGCAGGATTTTCATG-3 ʹ  Reverse: 5ʹ-TCAAGTGGCATAGATGTGGAAGAA-3 ʹ |
| *Reg3g* | Forward: 5ʹ-ATGCTTCCCCGTATAACCATCA-3 ʹ  Reverse: 5ʹ-GGCCATATCTGCATCATACCAG-3 ʹ |

# Supplementary Table 2. Antibodies used for flow cytometry analysis.

| **Antibody** | **Fluorophore** | **Concentration** | **Clone** | **Company** | **Cat. No.** |
| --- | --- | --- | --- | --- | --- |
| CD45 | BV510 | 1:300 | 30-F11 | BD Biosciences | 563891 |
| CD3e | Alexa 700 | 1:300 | 500A2 | BD Biosciences | 557984 |
| CD4 | FITC | 1:300 | RM4-5 | BD Biosciences | 561104 |
| CD17a | APC | 1:200 | TC11-18H10.1 | BioLegend | 506915 |
| Ly6G | PE | 1:200 | 1A8 | BD Biosciences | 561104 |

**
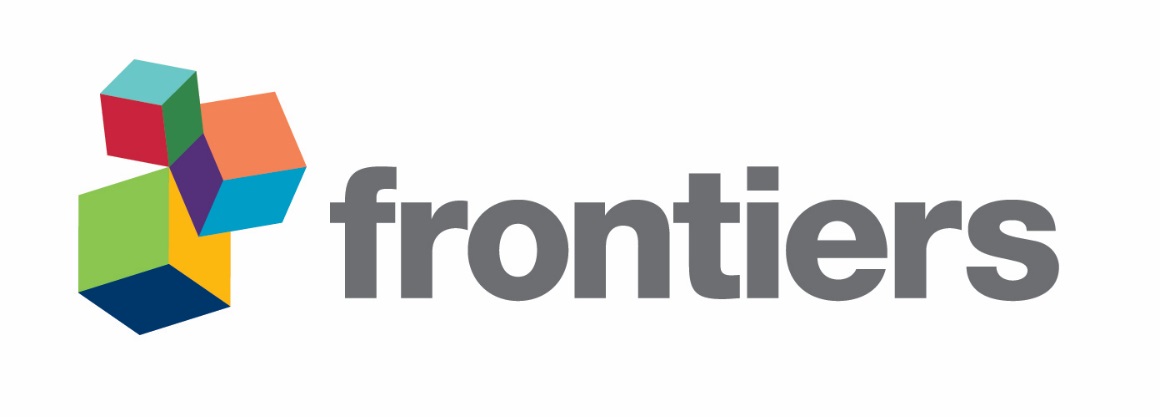
**
